# How Does Organizational Toxicity Affect Depression? A Moderated Mediation Model

**DOI:** 10.3390/ijerph20053834

**Published:** 2023-02-21

**Authors:** Ömer Akgün Tekin, Engin Üngüren, Ayşegül Doğrucan, Sevcan Yıldız

**Affiliations:** 1 Department of Gastronomy and Culinary Arts, Manavgat Faculty of Tourism, Akdeniz University, Antalya 07600, Turkey; 2 Department of Business Management, Faculty of Economics, Administrative and Social Sciences, Alanya Alaaddin Keykubat University, Antalya 07450, Turkey; 3 Department of Philosophy, Faculty of Letters, Akdeniz University, Antalya 07600, Turkey; 4 Department of Tourism and Travel Services, Social Sciences Vocational School, Akdeniz University, Antalya 07600, Turkey

**Keywords:** organizational toxicity, burnout, depression, occupational self-efficacy

## Abstract

Organizational toxicity is a key organizational issue today, impacting the success of both employees and organizations negatively alike. Negative working conditions revealed by organizational toxicity pave the way for an organizational atmosphere to arise, which negatively influences the physical and psychological well-being of employees, causing burn-out syndrome and depression. Thus, organizational toxicity is observed to have a destructive impact on employees and can threaten the future of companies. In this framework, this study examines the mediating role of burnout and moderator role of occupational self-efficacy, in the relationship between organizational toxicity and depression. Conducted as cross-sectional, this study adopts a quantitative research approach. To that end, convenience sampling was used to collect data from 727 respondents who are employed at five-star hotels. Data analysis was completed with SPSS 24.0 and AMOS 24 packages. Consequent to the analyses, organizational toxicity was determined to have a positive effect on burnout syndrome and depression. Moreover, burnout syndrome was found to have a mediating effect on the relationship between organizational toxicity and depression. In addition, occupational self-efficacy was found to have a moderator role on the effect of employees’ burnout levels on their depression levels. According to the findings, occupational self-efficacy is an influential variable on reducing the impact that organizational toxicity and burnout have on depression.

## 1. Introduction

By caring most about profitability and putting other organizational conditions into the background for a long time, organizations today strive to attract and retain talent. However, negative workplace conditions might render such efforts futile [1]. Accordingly, many studies have been conducted to examine negative workplace conditions and their impact on both the organization and employees [2]. To be more specific, a widely studied topic within the context of workplace negativity has been “organizational toxicity” (OT). The concept of “toxicity”, as the underlying factor of OT, is defined as “the inherent capacity of a substance to produce a harmful effect on the organism” [3]. While the term is heavily used in chemistry to describe the poisonous nature of matters [4], management studies, since the 1980s, have also been using terms such as “toxic” or “toxicity” to describe organizations [5,6]. Making ground in management studies as “organizational/workplace toxicity”, this concept refers to “a situation that causes employees to suffer and have problems, reduces interest in their jobs by negatively affecting their morale and motivation” [7]. OT can be accepted as workplace negativity, reducing overall employee satisfaction and performance, damaging teamwork and causing higher turnover rates [8]. From this perspective, OT appears to be an organizational problem, affecting employees in negative ways and, consequently, representing critical risks for the future of the organization.

“Toxic emotions” pave the way for the emergence of OT. They are consequences of “toxic events” in organizations [9]. Workplace negativities in organizations such as overworking, injustice, mobbing, infidelity, insensitivity, intrusiveness, lack of empathy and emotional intelligence and bullying can be examples of toxic events [7]. In fact, toxic events can be observed in all organizations on some level; however, if they are ignored, employees can leave behind innovative ideas and productivity [10]; their ties with the organization may be damaged, and they can experience issues such as anxiety, stress, depression and burnout syndrome (BS) due to the negative effects on their physical and psychological well-being [11,12,13]. Viewed as a problem caused by OT, BS is, at its core, a dynamic process, comprising (1) exhaustion, (2) depersonalization and (3) low self-esteem or personal accomplishment [14,15]. In addition, BS can be defined as a physical and emotional exhaustion syndrome, causing employees to have negative attitudes towards their jobs, while their desire for engagement is reduced [16,17]. Those who experience BS feel “helpless, hopeless, and powerless” [18]. Stress caused by OT in the relationship between OT and BS has a critical role. That is because stress is one of the destructive consequences of OT on employees [13,19]. The lengthy persistence of stress is one of the most important reasons for the emergence of BS [20]. Actually, Schaufeli and Enzmann [21] state that burnout is the end product of chronic stress due to work. Various studies in literature [22,23,24,25] support these arguments, revealing that OT positively affects BS.

BS is not only a negative effect of OT on employees [12]. BS is, at the same time, one of the key reasons for depression [26]. BS and depression are sometimes used interchangeably due to their similarities. While there are studies in the literature revealing that BS causes depression, there are also studies stating that depression causes BS. However, some studies state that BS and depression are very similar phenomena and overlap. In this respect, the findings regarding the relationship between BS and depression are inconsistent [27]. Maslach and Schaufeli [28] reported that one of the components of BS is depression. However, Hallsten [29] defined BS as a type of depression resulting from the exhaustion process. Bianchi et al. [30] stated that BS and depression exhibit a very high correlational relationship, and they revealed that the two variables overlap. Similarly, Bianchi and Brisson [31] pointed out that BS and depression overlap at the symptom and etiological level. In this study, the approach that accepts that BS causes depression was adopted. Because still, there is growing consensus in literature, arguing that BS must be considered as a specific problem in and of itself, separate from depression [32,33]. According to Glass and McKnight [34], there is a relationship between BS and depression that is far from the exact overlapping of the concepts. BS and depression have a similar relationship with control. This is because perceived lack of control causes burnout, which in turn leads to depressive symptomatology. Srivastava and Tang [26] have shown that BS causes increased health problems and depression, along with decreased performance of individuals. Demir [35] has determined that negative effects arising from leader–member interaction and stress, as well as burnout, are among the factors that cause depression. Depression is defined as a serious disorder with symptoms persisting for more than two weeks, such as sadness, loss of interest, loss of appetite, insomnia, fatigue, feeling worthless or guilty, difficulty in thinking and considerations of suicide [36]. Observed commonly in middle-aged individuals, depression [37] is accepted as a disabling psychiatric illness with personal and economic consequences [38]. Depression causes employees to have reduced productivity and [39] performance [40], display absenteeism behavior, short-term disability [41], become unemployed due to high turn-over rates [42] and receive lower incomes [39]. Depression is also a significant issue with respect to the burden it unloads on the global economy. It has been found that anxiety and depression cause an estimated loss of 1 trillion USD in global productivity [43].

Due to the positive relationship between BS and depression [44], BS is thought to influence the development of depression. Freudenberger [14] even claims that employees experiencing BS “look, act and seem depressed”. In addition, various studies in the literature [26,35,45,46] clearly reveal the positive relationships between BS and depression. 

Irrespective of the negative effect BS has on depression, varying studies in the literature point out that negative circumstances, arising from OT, may cause depression by themselves. In other words, while BS is one of the reasons employees experience depression [35], circumstances caused by OT are viewed as important factors by themselves for the development of depression. In this context, Danaher [47], Mohamed et al. [48], Rasool et al. [49] and Wang et al. [13] shared noteworthy findings, indicating that OT has a positive impact on depression. Moreover, Appelbaum and Roy-Girard [50] underline that employees in toxic organizations may find themselves in hopelessness, anger, low morale, poor communication and depression due to such circumstances, causing them to reveal poorer job performances and higher levels of absenteeism. Combining such findings in the literature with the ones stating that OT causes BS [22] and BS causes depression [35], we may hypothesize that BS plays a mediator role in the relationship between OT and depression. 

Defined as the individual’s faith in their talents to complete a particular task or overcome faced challenges [51], occupational self-efficacy (OSE) might be an aiding function to cope with certain issues arising from working conditions. While various studies in the literature [26,35] show that BS leads to depression, employees who feel occupational self-efficacy may experience lower levels of depression. In other words, OSE may function as a buffer for BS-caused factors to drag individuals into depression [52]. In this context, numerous studies in the literature [53,54,55,56] show negative relationships between BS and OSE. Alongside such negative relationships between BS and OSE, findings from past studies concerning the relationship between OSE and depression are important for the sake of this study. For example, Gecas [57] argues that OSE is a key factor for mental health, revealing that it specifically has a diminishing effect on depression. As cited in Gecas [57], the learned helplessness theory states that individuals feel inefficient when they believe that their actions have no consequences whatsoever on their surroundings, which can lead to depression. However, the exact opposite, namely the belief that their actions can have meaningful consequences can lead to possessing feelings of efficacy, which could be a barrier between them and depression. According to Manhas and Bakhshi [58], occupational self-efficacy of employees is an important resource for success and self-confidence. These people approach challenging conditions as matters to overcome, rather than threats. In this context, employees with high levels of self-efficacy would be more resilient against stress and depression, achieving better results. In other words, OSE acts as a buffer mechanism for preventing depression. 

**Figure 1:**
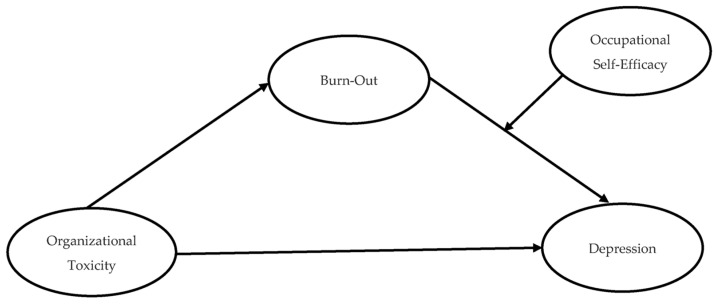
Conceptual model.

The purpose of this study is to examine the mediating role of burnout and the moderating role of occupational self-efficacy in the relationship between organizational toxicity and depression. The study aimed to test the moderated mediation research model shown in Figure 1. In this regard, based on the above literature review, the following hypotheses were proposed.

**Hypothesis** **1.** *Organizational toxicity positively affects depression.*

**Hypothesis** **2.** *Organizational toxicity positively affects burnout.*

**Hypothesis** **3.** *Burnout positively affects depression.*

**Hypothesis** **4.** *Burnout has a mediating role in the effect organizational toxicity has on depression.*

**Hypothesis** **5.** *Occupational self-efficacy has a moderating role in the effect burnout has on depression.*

**Hypothesis** **6.** *Occupational self-efficacy has a moderating role in the indirect effect organizational toxicity has on depression as mediated by burnout.*

In the literature review, it was seen that there are various studies focusing on organizational toxicity, workplace toxicity and burnout syndrome. In addition, it has been determined that there are many studies in the literature focusing on the relationships between burnout syndrome and depression. However, no studies examining organizational toxicity, burnout syndrome and depression variables in the context of the mediating role of burnout syndrome and the moderating role of self-efficacy could be accessed. In this respect, we believe that this study makes a theoretical contribution to the workplace toxicity literature and to researches in the field of tourism. In this study, we determined that occupational self-efficacy has a critical role in combating burnout syndrome and depression, which is affected by organizational toxicity. We consider that this finding can make a practical contribution to the managerial practices of tourism businesses specifically.

## 2. Materials and Methods

### 2.1. Research Design

This research study is a cross-sectional one, conducted with a quantitative research method. The population for the study comprises employees working at five-star hotels in Alanya and Manavgat, Turkey. The majority of the total bed capacity of the hotels in Alanya and Manavgat are in five-star hotels [59]. Convenience sampling from amongst non-probability sampling methods was selected for this study to reach out to a larger number of respondents. Data were acquired from five-star hotel employees in September and October of 2022. Having a minimum of three months experience at the current employer was one of the inclusion criteria. Another inclusion criterion was working full-time. The justification for this criterion is the idea that full-time employees spend more time at their workplace and hence can better evaluate the policies and practices of management. Data were collected from 16 different five-star hotels. After the hotels were identified, their general managers and human resources managers were contacted and informed about the nature and methods of the study to ensure their support. Two different methods were used to collect data. First, the face-to-face method was used, where employees were explained the aim and content of the questionnaire. The forms were then distributed to the respondents and collected back. This method allowed the collection of 456 questionnaires. The second method was the drop and collect method. To that end, human resources managers of the hotels were sent the questionnaires in closed envelopes. Respondents were given the questionnaires from these envelopes in the human resources department. A few days later, questionnaires were delivered back to human resources in closed envelopes again. These hotels were revisited after a couple of weeks to collect the questionnaires. A total of 362 questionnaires were collected out of the 500 that were left for the drop and collect method. In total, 818 questionnaires were collected, while 34 were empty and 57 were filled in erroneously, which is why 91 questionnaires were excluded from the analysis. Analyses were conducted with data from the remaining 727 respondents. In both methods, all respondents were informed about the voluntary nature of their participation, orally and in writing. All respondents remained anonymous, and they were reassured about the confidentiality of the data. G*Power software was used to calculate the sample size required for the assessed research model. It was then found that a minimum of 207 respondents were required to conduct the appropriate regression analysis for the research model. In line with the recommendations of Ring et al. [60], the inclusion of three times the number of respondents than the initially calculated number strengthens the adequacy of the research model. In this context, 727 respondents are enough to test the research model. Since two data collection methods were used in the research study, a *t*-test was conducted to determine whether or not respondents’ answers differed. Consequent to the *t*-test analysis, no significant differences were observed between data collected with two different methods. To prevent common method variance (CMV), a set of procedural and statistical methods were also adopted [61]. Procedurally, all respondents were assured that their responses were to be kept confidential and their anonymity was preserved. In the literature, anonymity assurance is used as a strategy to reduce social desirability bias [62]. Furthermore, independent, moderator and dependent variables were distributed randomly in the questionnaire with respect to procedure.

### 2.2. Measures

Data were collected via questionnaires. Seven statements, adapted from the perceived organizational toxicity scale developed by Kasalak and Aksu [63], were used for measurement of the independent variable. Cronbach’s alpha was found to be 0.93 in the original study. A high score from the scale indicates that boundaries of politeness are surpassed in interpersonal communication, and employees are undermined and exposed heavily to offensive words and actions. It also indicates a workplace where gossip and jealousy are frequently observed in behavior. The mediating variable of the study, burnout, was measured with the 10-item Burnout Measure Short Version (BMS), adapted by Malach-Pines [64]. Malach-Pines [64] adapted BMS with the purpose of establishing a convenient measurement tool, comprising 10 items, to fulfill the needs of both researchers and practitioners. Ease of use and high face validity make BMS a viable option for researchers. Calculated with data acquired from different ethnicities, professions and student groups, the scale is reported to have internal consistency coefficients ranging between 0.85 and 0.92 [65]. The Turkish validity and reliability study of the scale was conducted by Tümkaya et al. [66] and Capri [65]. Tümkaya et al. [66] reported an internal validity reliability coefficient of 0.91 with test-retest reliability of 0.70; while, Capri reported 0.91 and 0.88, respectively [65]. The moderator variable of the study, occupational self-efficacy, was measured with A Short Version of the Occupational Self-Efficacy Scale comprising 6 propositions, as developed by Rigotti et al. [67]. A high score from the scale reflects a high level of occupational self-efficacy. Üngüren and Tekin [68] adapted the scale into Turkish and reported an internal validity reliability coefficient of 0.96. The independent variable, depression, was measured with The Short Depression-Happiness Scale (SDHS), developed by Joseph et al. [69]. SDHS is a short and one-dimensional scale, comprising a total of 6 items with 3 negative and 3 positive statements to reduce response bias. The Turkish adaptation, validity and reliability study of the scale was conducted by Sapmaz and Temizel [70] and Yıldırım and Belen [71]. Both studies reported an internal consistency coefficient of 0.80. Perceived organizational toxicity, burnout and depression–happiness scales were marked on a five-point Likert-type scale (1 = Never, 5 = Always). Occupational Self-Efficacy Scale items were encoded from 1 = “strongly disagree” to 5 = “strongly agree”. The final section of the questionnaire includes questions on the demographics of respondents such as age, gender and education.

### 2.3. Statistical Analysis

Collected data were analyzed with the help of SPSS 24.0 (IBM, Armonk, NY, USA) and AMOS 24 (IBM, Armonk, NY, USA) packages. Before the analysis, the data were first scanned for missing values. No missing values were determined. Afterwards, normal distribution was checked in data. To that end, skewness and kurtosis values were analyzed. Three statistical analyses were conducted for the study to pinpoint the existence of issues via common method bias (CMB). Harman’s single factor analysis was first used. Then, CMB and variance inflation factor (VIF) values were checked. Finally, an alternative model was used to compare the research model by collecting all scales under one factor to test whether or not CMB represents a risk. The descriptive analysis of demographic variables was examined with frequencies and percentages. The test for the measurement model, on the other hand, utilized confirmatory factor analysis (CFA) to assess convergent and discriminant validity. Correlations between variables were analyzed with the use of the Spearman correlation coefficient. To test the mediation model and investigate the causal relationships among the main variables, analysis of covariance structure was used. The moderating role of employees’ occupational self-efficacy in the indirect effect of organizational toxicity on depression through burnout was also examined. For this purpose, the moderation mediation model was evaluated based on the moderation mediation index proposed by Hayes [72].

## 3. Results

### 3.1. Demographic Characteristics of Study Participants

Table 1 includes the demographics of respondents. In total, 64% of the respondents are men and 36% are women. More than half of them (62%) are single employees. With respect to the level of education, 46% are high school graduates, 32% are elementary school and 22% are university graduates. Respondents appear to be gathered around two age groups. While respondents in the 18–27 and 28–37 age groups represent 71% of the total, 5% are of age 58 and above. Moreover, 37% of the respondents have been working at their current place of employment for 1–3 years; 34% have been for 4–6 years; 16% for 7–9 years; and 13% for 10 years or more. A substantial proportion of the respondents who participated in the study work in restaurant and bar divisions (32%), housekeeping (20%), technical services (12%), kitchen (11%) and front office (10%).

### 3.2. Assessment of the Measurement Model

The measurement model was tested with confirmatory factor analysis (CFA) to determine the validity and reliability of the theoretical constructs. Table 2 shows the results of CFA, conducted to test the measurement model. Table 2 also shows the standardized factor loading and t-value for each item, as well as Cronbach’s α values and skewness and kurtosis values. Factor loads of all items are >0.50, while all factor loads are statistically (*p* < 0.001) significant [73]. Overall, the goodness-of-fit statistics for the model (χ^2^ [367, n = 727] = 798,378; *p* < 0.05; χ^2^/df = 2,175; RMSEA = 0.040; SRMR = 0.031; NFI = 0.939; RFI= 0.933; IFI = 0.966; TLI = 0.962; CFI = 0.966) show that the measurement model is an acceptable one [74]. Skewness and kurtosis values of scale items were found to be between +1.5 and −1.5, which indicates normal distribution for the data in the study [75]. Moreover, Cronbach’s α values for organizational toxicity, burnout, depression and occupational self-efficacy were calculated to be 0.896, 0.913, 0.892 and 0.908, respectively. All scales have α > 0.70, which points to the existence of their internal consistency [74].

Data regarding the convergent and discriminant validities of the measurement model are presented in Table 3. Each item’s factor-loading values were >0.5, AVE values were greater than 0.50, CR values greater than 0.70, and AVE values were less than CR values, which indicates the convergent validity of factors [76]. To test discriminant validity, the criterion recommended by Fornell and Larcker [77] and HTMT coefficients recommended by Henseler et al. [78] were used (Table 3). Furthermore, MSV and ASV values were calculated. According to the criteria from Fornell and Larcker [77], the square root of AVE (√AVE) of each variable should be greater than the correlation coefficients among all variables, and AVE values of scales should be greater than MVS and ASV to indicate discriminant validity [70], which was proven for this study. On the other hand, HTMT values, as recommended by Henseler et al. [78], were less than 0.90, revealing that scales have discriminant validity. The results of intervariable correlation within the scope of this study are displayed in Table 3. Accordingly, a positive and significant relationship was found between organizational toxicity and burnout (r^2^ = 0.59, *p* < 0.001) and depression (r^2^ = 0.44, *p* < 0.001), while no significant relationship was identified between organizational toxicity and organizational self-efficacy (r^2^ = −0.07, *p* > 0.05). A positive relationship was found between burnout and depression (r^2^ = 0.55, *p* < 0.001), and a negative relationship was found between occupational self-efficacy and depression (r^2^ = −0.50, *p* > 0.001).

Certain procedural solutions were also adopted to reduce the risk of common method variance (CMV) [61]. First of all, respondents were informed about the scientific aim of the study, the voluntary basis for participation, the fact that there are no right or wrong answers, the confidentiality for responses, as well as the reassurance that their information will not be shared with any third parties at all. Furthermore, no spaces for names or last names were left on the questionnaire. Explanations were also provided that hotel management approved employees’ participation in the study. Following the procedural steps, statistical tests were conducted to identify CMV. First, Harman’s single-factor test was applied. Harman’s single-factor test is one of the methods used to test for common method variance bias. The basic assumption of Harman’s single-factor test is that no single factor should explain more than 50% of the variance [61]. Consequent to the factor analysis, four factors were identified with eigenvalues over 1 and these four factors explained 63% of the total variance. The first factor accounts for 20% of the variance. Since no single factor emerged and the first factor did not account for most of the variance, there is no serious CMB for this study. [79]. Second, CFA was conducted, where all questionnaire items are collected under one factor. According to the results in Table 4, the goodness-of-fit indices of the single-factor model are significantly weaker than those of the research model. These results show that CVM does not represent an issue for this study. In addition, it was tested for multicollinearity between the variables. The tolerance and variance inflation factor (VIF) values were used as indicators in the multicollinearity test. All variables were found to have VIF values, ranging between 1.55 and 1.01. These values are below the designated threshold, indicating that multicollinearity was not a bias problem in the present data.

### 3.3. Testing the Hypotheses

The hypotheses were tested using IBM AMOS 24.0 and path analysis was performed with the maximum likelihood method. Bias-corrected bootstrapping with 5000 resamples and 95% confidence intervals (CI) was used to estimate the indirect effects of the mediator variables. First of all, the total, direct and indirect effects on the relationship ORGTOX → BRNT → DPRSYN were tested without the moderator variable. To test H_1_ (ORGTOX → DPRSYN), a structural latent variable model was tested in which organizational toxicity is exogenous and depression is endogenous. The goodness of fit statistics for this hypothesis (χ^2^ [60, n = 727] = 145.80; *p* < 0.01; χ^2^/df = 2430; RMSEA = 0.044; SRMR = 0.029, NFI = 0.973; RFI = 0.965; IFI = 0.984; TLI = 0.979; CFI = 0.984) indicate that the measurement model is an acceptable model [74]. According to the results of path analysis in Table 5, ORGTOX has a significant and positive influence on DPRSYN (β = 0.48, *p* < 0.001). Thus, H_1_ (ORGTOX → BRNT) is supported. In order to test the other hypotheses of the study, a separate model was constructed in which burnout was a mediating variable. The goodness-of-fit statistics (χ^2^ [225, n = 727] = 556.466; *p* < 0.01; χ^2^/df = 2.473; RMSEA = 0.045; SRMR = 0.032, NFI = 0.943; RFI = 0.936; IFI = 0.765; TLI = 0.961; CFI = 0.965) obtained for this model are within the threshold values, indicating that the model is compatible and acceptable with the data. According to the results of path analysis, organizational toxicity (ORGTOX → BRNT) significantly and positively predicts burnout (β = 0.65, *p* < 0.001). Burnout, which is a mediating variable, (BRNT → DPRSYN), also significantly and positively affects depression (β = 0.51, *p* < 0.001). These findings support Hypotheses 2 and 3. 

A path analysis based on the bootstrap method was used to test whether burnout plays a mediating role (ORGTOX → BRNT → DEPRSYN) in the relationship between organizational toxicity and depression. According to the bootstrap results in Table 6, the indirect effect of organizational toxicity on depression through burnout is significant [β = 0.33, %95 BCA CI (0.26; 0.40)]. This result shows that burnout mediates the relationship between organizational toxicity and depression. In other words, employees who are exposed to organizational toxicity perceive higher levels of burnout, and therefore suffer from higher levels of depression. This finding also supports Hypothesis 4.

Finally, the model for the moderating role of OCCEFCY in the indirect effects of ORGTOX on DPRS through BRNT was examined. Therefore, the moderation mediation model was evaluated on the basis of the moderation mediation index proposed by Hayes [72]. To determine the values of the *t*-test, a bootstrap resampling technique was used to account for 5000 subsamples. Firstly, the moderator role of occupational self-efficacy in the effect of burnout on depression was examined. According to the results of the analysis in Table 7, BRNT has a positive effect on depression (β = 0.57, %95 [0.51; 0.62], *p* < 0.001), and OCCEFCY has a negative effect (β = −0.42, %95 [−0.47; −0.38], *p* < 0.001). Interaction effect value (OCCEFCY × BRNT), which shows the existence of a moderator effect, is found to be significant [β = −0.11, %95 (−0.16; −0.05), *p* < 0.001], pointing out that occupational self-efficacy has a moderator effect. These results indicated that the relationship between BURNT and DPRSYN is moderated by OCCEFCY. The effect of burnout on depression is greater for employees with low levels of perceived occupational self-efficacy (β _OCCEFCY(High)×BRNT→DPRSYN_ = 0.68, %95 CI [0.61; 0.76]; (β _OCCEFCY(Low)×BRNT→DPRSYN_ = 0.46, %95 CI [0.37; 0.54]). As employees’ professional self-efficacy increases, the effect of burnout on depression partially decreases. These results suggest that as employees’ occupational self-efficacy increases, the negative effect of burnout on depression is buffered. These results support Hypothesis 5.

**Figure 2:**
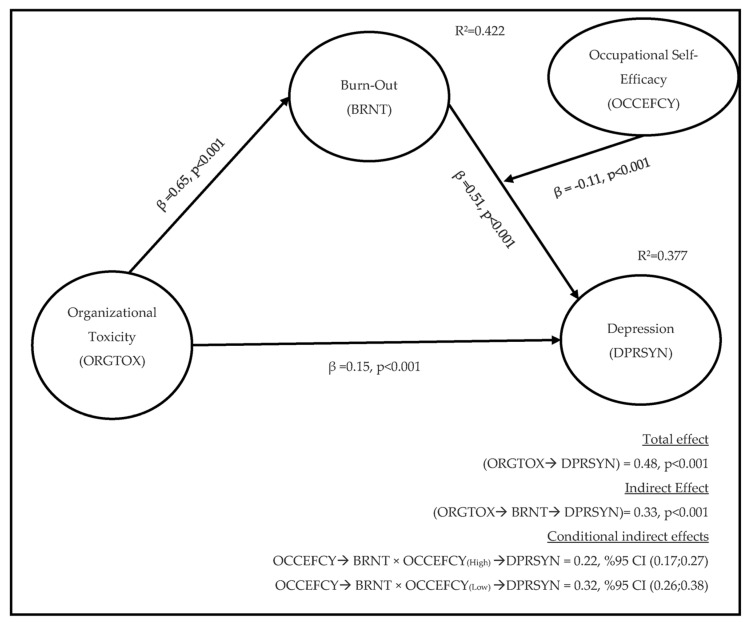
Results of the conceptual model.

The final step was the examination of the moderating role of OCCEFCY in the indirect effect of OCCEFCY on DPRSYN through BRNT. According to the result in Table 8, the index of moderated mediation (β = −0.05, %95 CI [−0.09; −0.02]) was found to be statistically significant. These results show that the indirect effect of organizational toxicity differs by the occupational self-efficacy of employees. When employees’ perceived occupational self-efficacy is low, the indirect effect of organizational toxicity (β_[OCCEFCY(High)]_ = 0.32, %95 CI (0.26; 0.38), β_[OCCEFCY(Low)]_ = 0.22, %95 CI [0.17; 0.27]) on depression through burnout, increases. The results suggest that occupational self-efficacy buffers the effect of organizational toxicity on depression through burnout. Figure 2 provides details of the parameter estimates for the model. Therefore, Hypothesis 6 was supported as well.

## 4. Discussion

Good working conditions are just as important as offered financial means for an organization to be successful. Conditions that cause workplace negativity within the organization negatively influence employees’ physical and psychological well-being, organizational loyalty, performance, motivation and effectiveness [2,11,12,13,80]. In this context, eliminating or improving such conditions that would lead to workplace negativity is quite important for both organizations and employees. This study focuses on the OT problem as it is one of the factors approached within the framework of workplace negativity [81]. Within the framework of the hypotheses, relationships between OT, BS and depression variables were investigated; in addition, we inquired about the moderating role of OSE in them. This section attempts to discuss, interpret and explain the results of the conducted hypotheses tests in comparison with the findings of other studies in the literature. Conservation of Resources (COR) theory, developed by Hobfoll [82], is used to explain the findings.

Within the scope of the study, Hypothesis 1 concerning the link between OT and BS was tested. Consequently, the hypothesis was supported as OT is observed to positively affect BS. Toxic organizations are known for their negative workplace conditions such as a history of poor decision-making, high levels of employee dissatisfaction, ineffective working conditions and destructive human relations [50,83]. Each one of these conditions can be viewed as a toxic event, causing toxic emotions [84]. The exposure to toxic events that cause OT [9] leads to long-term stress [85]. Persisting stress is a key reason underlying BS [20]. In this context, acquired results appear to be in line with the theoretical assessments of previous studies. Moreover, a study conducted by Jaime et al. [86] on psychiatrists found that toxic management conditions cause toxic feelings in employees, which positively affects BS. Rusbasan et al. [87] conducted a study on student athletes, where they found that toxic coaching by their coaches can lead athletes to experience BS. Ghanbari and Mojooni [88] concluded in their study that toxic leadership causes teachers to suffer from BS. Another study, conducted by Koropets and Polents [89], underlines that toxic working conditions in organizations lead to BS, while employees’ ways of making sense of the toxic conditions are also influential in causing such a phenomenon to occur. On the other hand, Bakan et al. [90] and Hadadian and Zarei [91] found in their studies positive links between stress and toxic leadership, which is considered to be one of the components of OT.

Consequent to the testing of Hypothesis 2, which concerns BS and depression, BS was found to influence depression positively, which supports the hypothesis. Within the framework of this hypothesis, it is of benefit to note the results of previous studies, revealing that BS and depression are separate concepts. Koutsimani et al. [92] conducted a meta-analysis where they examined the studies on “burnout and depression” and “burnout and anxiety” links, published between 2007 and 2019. Consequently, their findings “revealed no conclusive overlaps between burnout and depression and burnout and anxiety, indicating that they are different and robust constructs.” Iacovides et al. [93] found in their study that a person suffering from depression cannot also suffer from BS simultaneously. Other studies in the literature can also be found on the distinction between BS and depression [21,94]. Many existing studies in the literature on the relationship between BS and depression reveal a positive link between them. Bianchi and Laurent [95] conducted a study with 54 human resources employees and used eye-tracking technology for their research. They found positive links between BS and depression. Shirom and Ezrachi [96] also conducted a study with 704 senior army officers, where they observed positive relations between participants’ levels of BS and depression. In a study conducted by Upadyaya et al. [97] where 1.415 employees were observed for two years within the context of occupational health, employees were found to reveal positive links between BS levels and depression. Meier and Kim [98] utilized meta-regression analyses in their study where they examined the relations between BS and depression. A total of 46.191 individuals participated in the study, while the authors focused on 69 studies. All examined studies identified positive correlations between BS and depression (overall effect size 0.492).

Hypothesis 3 concerns the positive effect of OT on depression, while Hypothesis 4 concerns the moderator role of BS on the effect OT has on depression. Consequent to the analyses, both hypotheses were observed to be supported. Anjum and Ming [99] point out that depression is one of the consequences of OT. In addition, Carlock [83] concluded in their study that respondents actually suffer from depression due to the toxic workplace conditions, despite having the motivation to succeed in their jobs, highlighting the relationship between OT and depression. Conducted by Rasool et al. [49] in Chinese bank employees, another study concluded that OT causes depression, while this negatively influences employees’ levels of productivity, leading to traumatic consequences for employees. Wang et al. [13] conducted a study with employees of renewable energy project-based companies, and concluded that OT causes depression, which becomes an important source of stress for the employees of such an organization. Besides being a serious implication of OT, stress is a key factor underlying BS, as highlighted by Maslach et al. [20]. BS is even viewed as the ultimate consequence of work-related chronic stress [21]. Furthermore, when results of studies examining the relations between BS and depression are reviewed, BS appears to be identified as a factor that causes depression [100]. For example, consequent to the study conducted by Demir [35] on teachers, BS was found to affect depression positively. The same study also notes that leader–member interaction reduces teachers’ stress and depression levels by reducing their levels of BS. Woo Kyeong [46] conducted a study with cyber-university students in Korea and identified positive relations between BS and depression. However, the study also argues that self-compassion mitigates the effect BS has on depression. In another study, carried out by Bakker et al. [100], it was underlined that BS and depression are separate, yet interrelated concepts. In the same study, Bakker et al. [100] stated that “burnout is a work-related phenomenon whereas depression is more pervasive and context-free in nature”. In this context, the stress that employees experience is observed to lead to depression, which may spread throughout their lives. In line with this evaluation, Bender and Farvolden [101] underline that depression is an important reason underlying employees’ exposure to severe stress. In this context, this finding shows that BS plays a mediator role in the relationship between OT and depression, which is in line with the findings of previous studies in the literature.

Hypothesis 5 posits that occupational self-efficacy plays a moderator role on the effect burnout has on depression, while Hypothesis 6 posits that occupational self-efficacy plays a moderator role on the indirect effect organizational toxicity has on depression through burnout. According to these findings, the effect BS has on depression changes as per employees’ OSE levels. In this context, it was determined that the effect of the burnout levels of employees, who find themselves adequate in occupational terms, on depression is lower than that of those who do not find themselves as such. In other words, high levels of occupational self-efficacy play a reductive and buffering role on the effect burnout has on depression. Shoji et al. [52] conducted a study with the meta-analysis method where they found that OSE acts as a preventative factor against the negativities stemming from burnout. In another study, conducted with 80 physicians, Aftab et al. [53] concluded that physicians with higher levels of OSE are less affected by burnout. Yang [102] also concluded, in a study which was conducted with 268 nurses working at a teaching hospital, that nurses with low levels of OSE are more affected by burnout. Findings acquired in this study reveal the moderator role of OSE in the relationship between BS and depression. On the other hand, depression is known to be closely related to poor self-efficacy [103]. This way, while improving OSE is a factor that reduces the effects of BS on one hand, it is evaluated as a reductive factor for depression. In this context, Hypotheses 5 and 6 reveal similar findings to those in previous studies that can be found in the literature.

We believe that the results of this study can be explained within the context of COR theory. According to COR, employees with poor personal resources are expected to lose some of their resources that may negatively influence their psychological well-being [82]. Thus, employees with lower levels of OSE levels are affected more by BS and depression, stemming from OT, which can be explained within the context of COR theory. 

While the study contributes significantly to a gap in the literature, specifically in terms of the discipline of tourism and organizational toxicity, it still has certain limitations, as is the case in all research endeavors. One of the most important limitations of the study is that it is a cross-sectional study. Additionally, scales used to collect data are in the form of self-reports, which may be deemed as another limitation. Moreover, data were collected only from respondents who work at five-star hotels in a particular region and in a particular season. In this context, the findings of the study are limited in terms of representing only the data from the season and employees in the relevant region. Within the framework of these limitations, some recommendations can be made for future studies. Adopting a longitudinal study approach for future research endeavors might contribute to acquiring stronger findings. On the other hand, utilizing up-to-date methods such as neuro-imaging and laboratory techniques for specifically the burnout variable in such studies may yield more objective results. To that end, measuring employees’ occupational self-efficacy levels through experimental methods may also bring about a whole new depth. Furthermore, working with larger-scale samples in future studies would help to generalize findings for larger masses.

## 5. Conclusions

Many studies can be found in the literature on the relationship between BS and depression. However, this study approaches the relationship between BS and depression in relation with OT as a variable, and examines the moderator role of OSE herein. From this perspective, the study focuses on a niche field. In this context, the results of the study can be claimed to shed light on a previously dark area in the literature. OT is a problem that arises when multiple factors causing workplace negativity come together [7]. To deal with this problem, toxic events that cause workplace negativity must be identified, which can be viewed as an important step in battling such factors. However, we also observe that developing additional methods to contribute to employees’ OSE levels is important in battling depression, which is listed among the potential consequences of OT. As determined through this study, the finding that OSE plays a moderator role in the relationship between BS and depression is quite interesting. This finding tells us that OSE plays a key role in the burnout state of employees who work in a toxic workplace, turning into depression. In other words, we have found in this study that investing in matters to improve employees’ OSE levels is instrumental in battling the consequences of OT to prevent employees in a toxic workplace from being dragged into depression.

## Figures and Tables

**Table 1:** Characteristics of the participants (n = 727).

Variable	Category	n	%
Gender	Female	260	36%
	Male	467	64%
Marital status	Single	452	62%
	Married	275	38%
Education	Elementary	230	32%
	High school	337	46%
	Bachelor’s degree	160	22%
Age	18–27	222	31%
	28–37	290	40%
	38–47	117	16%
	48–57	63	9%
	58 and over	35	5%
Tenure	1–3 years	267	37%
	4–6 years	249	34%
	7–9 years	117	16%
	10 years and over	94	13%
Department	Restaurant and bar	234	32%
	Kitchen	77	11%
	Housekeeping	148	20%
	Front office	75	10%
	Accounting	34	5%
	Sales and reservation	28	4%
	Technical service (maintenance)	87	12%
	Other	44	6%

**Table 2:** Result of the measurement model.

Variables	Items	Factor Loadings	T-Value	Skewness	Kurtosis	Cronbach’s α
Organizational Toxicity	ORGTOX1	0.804	Fixed	0.50	−0.23	0.896
	ORGTOX2	0.706	19.71 ***	−0.29	−0.94	
	ORGTOX3	0.623	17.43 ***	−0.26	−0.22	
	ORGTOX4	0.694	19.71 ***	−0.19	−0.44	
	ORGTOX5	0.739	21.42 ***	0.18	−0.48	
	ORGTOX6	0.798	23.56 ***	0.23	−0.43	
	ORGTOX7	0.825	24.47 ***	0.40	−0.32	
Burnout	BRNT1	0.705	Fixed	0.44	0.26	0.913
	BRNT2	0.688	17.54 ***	−0.21	−0.95	
	BRNT3	0.675	17.37 ***	−0.26	−0.22	
	BRNT4	0.705	18.05 ***	−0.31	−0.55	
	BRNT5	0.676	17.50 ***	−0.17	−0.25	
	BRNT6	0.661	17.03 ***	−0.08	−0.31	
	BRNT7	0.724	18.59 ***	−0.09	−0.54	
	BRNT8	0.753	19.30 ***	−0.01	−0.58	
	BRNT9	0.788	20.29 ***	0.19	−0.36	
	BRNT10	0.827	21.19 ***	0.39	−0.07	
Depression	DPRSYN1	0.779	Fixed	0.39	−0.40	0.892
	DPRSYN2	0.712	17.93 ***	−0.21	−0.71	
	DPRSYN3	0.723	20.18 ***	−0.16	−0.54	
	DPRSYN4	0.760	21.62 ***	−0.21	−0.38	
	DPRSYN5	0.804	23.14 ***	−0.07	−0.50	
	DPRSYN6	0.842	24.42 ***	−0.03	−0.25	
Occupational Self−Efficacy	OCCEFCY1	0.844	Fixed	0.03	−0.37	0.908
	OCCEFCY2	0.823	27.60 ***	0.22	−0.50	
	OCCEFCY3	0.802	26.31 ***	−0.19	−0.83	
	OCCEFCY4	0.782	25.40 ***	−0.15	−0.85	
	OCCEFCY5	0.663	19.48 ***	0.01	−0.79	
	OCCEFCY6	0.872	29.31 ***	0.10	−0.83	

*** *p* < 0.001.

**Table 3:** Correlations, convergent and discriminant validity of observed variables.

	1	2	3	4	CR	AVE	MSV	ASV	HTMT Analysis			
									1	2	3	4
1. ORGTOX	[0.74]				0.90	0.55	0.42	0.18				
2. BRNT	0.59 **	[0.72]			0.92	0.52	0.42	0.22	0.66	-	-	-
3. DPRSYN	0.44 **	0.55 **	[0.77]		0.90	0.60	0.37	0.25	0.50	0.62	-	-
4.OCCEFCY	−0.07	−0.03	−0.50 **	[0.80]	0.91	0.64	0.29	0.09	0.08	0.03	0.55	-

ORGTOX: Organizational Toxicity, BRNT: Burnout, DPRSYN: Depression, OCCEFCY: Occupational Self-Efficacy, CR = Composite Reliability, AVE = Average Variance Extracted, ASV = Average Shared Variance, MSV = Maximum Shared Variance, [] = The square root of the AVE. (√AVE), ** *p* < 0.01.

**Table 4:** Comparison of the goodness-of-fit values of the research model with single factor structure.

Models	X²	df	X²/df	CFI	SRMR	RMSEA	Model Comparison		
							∆X²	∆df	*p* (∆X²)
1. Hypothesized model ^a^	798.38	367	2.175	0.966	0.031	0.04	-	-	
2. One-factor Model ^b^	6394.2	377	16.985	0.526	0.157	0.148	5595.83	10	0.000

^a^ = Organizational Toxicity; Burnout; Depression; Occupational Self-Efficacy. ^b^ = Organizational Toxicity + Burnout; + Depression + Occupational Self-Efficacy.

**Table 5:** Result of the structural model.

Hypothesis	Relation			β	SE	T Values	*p*	R^2^
Hypothesis 1	ORGTOX	→	DPRSYN	0.48	0.036	11,557	<0.001	0.230
Hypothesis 2	ORGTOX	→	BRNT	0.65	0.035	14,536	<0.001	0.422
Hypothesis 3	BRNT	→	DPRSYN	0.51	0.056	9440	<0.001	0.377
	ORGTOX	→	DPRSYN	0.15	0.040	3323	<0.001	

**Table 6:** Results of mediation analysis.

Hypothesis	Relation	Indirect Effect			Relation	Direct Effect		Total Effect	
		β	LLCI	ULCI		β	*p*	β	*p*
Hypothesis 4	ORGTOX → BRNT → DPRSYN	0.33	0.26	0.40	ORGTOX → DPRSYN	0.15	<0.001	0.48	<0.001

**Table 7:** Moderation analysis result.

Hypothesis 5	β	T Values	*p*	LLCI	ULCI
BRNT → DPRSYN	0.57	21.01	*p* < 0.001	0.51	0.62
OCCEFCY → DPRSYN	−0.42	−18.85	*p* < 0.001	−0.47	−0.38
OCCEFCY × BRNT → DPRSYN	−0.11	−3.74	*p* < 0.001	−0.16	−0.05
OCCEFCY(_High)_ × BRNT → DPRSYN	0.68	10.85	*p* < 0.001	0.37	0.54
OCCEFCY(_Low)_ × BRNT → DPRSYN	0.46	17.33	*p* < 0.001	0.61	0.76

**Table 8:** Moderated mediation analysis.

Hypothesis	Relations	Mediator	Moderator	Index of MM	SE	LLCI	ULCI
Hypothesis 6	OCCEFCY → BRNT × OCCEFCY → DPRSYN	BRNT	OCCEFCY	−0.05	0.01	−0.09	−0.02
OCCEFCY → BRNT × OCCEFCY_(Low)_ → DPRSYN		BRNT	OCCEFCY	0.32	-	0.26	0.38
OCCEFCY → BRNT × OCCEFCY_(High)_ → DPRSYN		BRNT	OCCEFCY	0.22	-	0.17	0.27

## Data Availability

The data presented in this study are available in anonymized form upon request from the corresponding author.
